# Interpersonal Liking Modulates Motor-Related Neural Regions

**DOI:** 10.1371/journal.pone.0046809

**Published:** 2012-10-05

**Authors:** Mona Sobhani, Glenn R. Fox, Jonas Kaplan, Lisa Aziz-Zadeh

**Affiliations:** 1 Neuroscience Graduate Program, University of Southern California, Los Angeles, California, United States of America; 2 Brain and Creativity Institute Los Angeles, Los Angeles, California, United States of America; 3 Department of Psychology, University of Southern California, Los Angeles, California, United States of America; 4 Division of Occupational Science and Occupational Therapy, University of Southern California, Los Angeles, California, United States of America; Ghent University, Belgium

## Abstract

Observing someone perform an action engages brain regions involved in motor planning, such as the inferior frontal, premotor, and inferior parietal cortices. Recent research suggests that during action observation, activity in these neural regions can be modulated by membership in an ethnic group defined by physical differences. In this study we expanded upon previous research by matching physical similarity of two different social groups and investigating whether likability of an outgroup member modulates activity in neural regions involved in action observation. Seventeen Jewish subjects were familiarized with biographies of eight individuals, half of the individuals belonged to Neo-Nazi groups (dislikable) and half of which did not (likable). All subjects and actors in the stimuli were Caucasian and physically similar. The subjects then viewed videos of actors portraying the characters performing simple motor actions (e.g. grasping a water bottle and raising it to the lips), while undergoing fMRI. Using multivariate pattern analysis (MVPA), we found that a classifier trained on brain activation patterns successfully discriminated between the likable and dislikable action observation conditions within the right ventral premotor cortex. These data indicate that the spatial pattern of activity in action observation related neural regions is modulated by likability even when watching a simple action such as reaching for a cup. These findings lend further support for the notion that social factors such as interpersonal liking modulate perceptual processing in motor-related cortices.

## Introduction

Observing goal-directed actions recruits a concert of neural resources. When we observe another person’s goal-directed motor actions, a rich array of information is read into the brain, giving us the necessary input to understand aspects of the observed action, including the possible outcomes of the action to the intent behind the action. Being able to gauge the intent of others’ actions is a vital skill that is highly relevant to the survival of the observer. In its most basic form, observing another person performing an action reveals a network of neural regions, including the inferior frontal gyrus (IFG), ventral premotor cortex (vPMC), and inferior parietal lobule (IPL) [1]. Specific motor-related neurons in the macaque monkey brain have been found to be active both when the monkey performs an action and when it observes the same or a similar action performed by another individual [2]. It has been suggested that these neurons are “mirroring” the actions that they observe, and that this may be a mechanism by which the monkey can simulate and understand these observed actions [3]. Neural regions exhibiting these properties in the macaque brain include the (F5) vPMC and the IPL. Functional neuroimaging studies in humans have provided a means for examining neural processing during action observation, revealing the recruitment of homologous regions to those found in the monkey brain (Gallese, Keysers, & Rizzolatti, 2004; Rizzolatti & Craighero, 2004). Evidence suggests that activation of human motor-related brain regions during the observation of actions is similar to activation when an action is performed. Observing a wide array of actions, ranging from object directed actions to communicative gestures [4]–[6] engages this fronto-parietal network.

Other investigations have revealed how these regions may be modulated by higher-level and socially relevant factors. For instance, emerging evidence suggests that these brain regions may be modulated by factors such as physical differences [7]–[9], and culture [5], [6]. In parallel, social group membership has been shown to modulate behavior [10] and physiological responses [11], leading into investigations of neural correlates of these observations. Findings from these investigations have revealed the effect of aspects of social group membership on various sensory-motor and cognitive processes. For example, Hart and colleagues (2000) demonstrated that both Black and White individuals displayed increased amygdala activation to out-group faces [12]. Another study revealed that Caucasian and Chinese individuals displayed decreased neural activation in the anterior cingulate cortex and inferior frontal/insula in response to viewing the application of painful stimulation to out-group members [13]. Thus, it appears that neural processing differs for in-groups and out-groups across various types of stimuli, particularly when group membership is defined by race.

It is unknown, however, how social group-based interpersonal liking, can affect sensory-motor neural regions, such as those involved in action observation. Generally, it has been demonstrated that action observation related neural regions are more active in response to stimuli of the self as opposed to that of others [14], [15], as well as for people who are more physically similar to oneself [5]. Based on these findings and the notion that members of one’s social group can be viewed as an extension of oneself [16], one would expect that action observation related neural regions would be differentially modulated by how we feel about the person we are observing–i.e. how much we like the person or whether or not they like us. Part of this assumption comes from the fact that individuals typically have more empathy for members of their own social group [17], and that the activity in action observation related neural regions has been shown to be correlated with scores on empathy scales [18], [19].

Thus, given these previous studies, one would expect that group membership would modulate activity in the MNS during action observation. A few studies have been conducted on this topic, with conflicting results. Using corticospinal excitability as a measure of motor system involvement in action observation, Molnar-Szakacs and colleagues (2007) found increased activity when watching members of the same social group performing culture-specific gestures [5], while Desy and Theoret (2007) found increased corticospinal excitability for viewing hand actions made by members of another race [8]. Using functional magnetic resonance imaging, Losin et al (2011) found enhanced activity in fronto-parietal regions during imitation of meaningless gestures performed by members of one racial outgroup (but not another) [20]. Another recent study discovered increased activity in the posterior parietal action observation-related region (IPL) and the insula in response to member’s of one’s own race performing communicative hand gestures [6]. Taken together, these studies indicate that how the brain shapes its response to observed actions is modulated by many factors, including social group membership and physical similarity to self.

These action observation studies, however, are limited by the fact that they confound social group membership with physical differences between ingroup and outgroup members. That is, in the previous studies, group membership is manifested by physically looking different than the observer’s ingroup, and thus it is unknown whether the observed effects are due to group membership or to physical similarity. Additionally, two of these action observation studies [5], [6] focus on gestures in a role of communication and culture, but do not address the more fundamental question of goal-directed action execution (e.g., raising a cup to the lips) outside of social communication. Lastly, these studies only address perception of group membership, but do not assess interpersonal liking that stems from in-group and out-group interactions. While a recent study exploring empathy for suffering has demonstrated that group membership independent of physical differences can modulate neural responses in neural regions such as the insula and nucleus accumbens [21], it is important to determine if the same is true for action observation and corresponding motor related neural regions.

A further limitation of previous work is that subject’s may feel that negative feelings about the out-group member are socially unacceptable, although they likely possess some unconscious biases [22]. This conflict manifests in the neuroimaging data as a complex time-dependent neural response, whereby neural regions involved in cognitive control of such feelings become active after the response is initially formed by limbic regions. For example, viewing an outgroup member’s face at shorter intervals initially causes increased amygdala activation, however, at longer stimulus presentation intervals, amygdala responses were dampened as frontal areas associated with cognitive control displayed increased activation [23]. To address these limitations in the literature, we use a simple object-directed action observation task and an interaction between two social groups which were: a) physically similar to one another (i.e. all Caucasian), and b) able to openly express dislike for the other group without being hindered by social stigma (i.e. it is not unreasonable for a Jewish individual to express dislike for a social group that openly hates and threatens their own social group, especially one that is commonly openly treated by disdain and contempt from the general population). With this study, we specifically aimed to investigate whether the liking or disliking of individuals which is derived from group membership, modulates regions involved in action observation. To address these goals, we recruited Jewish males and presented to them biographies of eight individuals’ lives, half of whom were presented as dislikable, neo-Nazis and half presented as likable, open-minded individuals. The participants then viewed these likable and dislikable individuals performing simple motor actions (e.g. reaching for, grasping, and bringing a water bottle to the lips) during a functional magnetic resonance imaging scan. Because we expect the overall effect size to be relatively modest (e.g., due to physical similarities between the likable and dislikable individuals) and traditional cognitive subtraction approaches may not be sensitive enough to disambiguate the two conditions, we used multivoxel pattern analysis (MVPA), in addition to typical univariate fMRI analysis, to investigate whether BOLD signal in brain regions previously implicated in action observation processes (inferior frontal cortices, inferior parietal cortices), would be able to distinguish liked persons from disliked persons during an action observation task. Because MVPA considers multiple voxels simultaneously, it is more sensitive than traditional univariate techniques that analyze each voxel separately [24], [25]. Furthermore, MVPA can reveal when the spatial pattern of activity changes between conditions even when the overall signal level does not differ [26], [27]. If, as previous studies have suggested, activity in action observation brain regions are modulated by higher level social factors, then we predict that these regions will exhibit different patterns of activity when observing liked and disliked individuals perform actions.

## Materials and Methods

### Participants

Nineteen healthy, Jewish males (18–30 years of age, mean ± SD = 21.9±3.58) participated in the experiment. Male participants were exclusively included in this study due to the fact that a previous study indicated that neural modulation caused by interpersonal liking may differ between genders, with males displaying more modulation in neural regions associated with shared representations when viewing someone they dislike [28]. Two participants had to be removed from all analyses due to technical issues; therefore, all discussed results involve the remaining 17 participants. Inclusion criteria included high scores on self-rating measures of Jewish identity using an ethnic identity measure (mean = 42.4, out of 48) [29], as well as a scale created to assess the participant’s self-reported affiliation with the Jewish religion (mean = 39, out of 48). All participants were right-handed, had normal or corrected-to-normal vision, and had no neurological or psychiatric history. Written informed consent was obtained from all participants before inclusion in the study. This study was approved by the University of Southern California’s Institutional Review Board and all research activities were performed in accordance with the Institutional Review Board’s policies.

General Procedure and Design: All subjects participated in a pre-scan training session where they were familiarized with eight individuals through the use of photos and corresponding fictional biographies. During this session, they completed several behavioral questionnaires on group identity, empathy and how much they liked the individuals they observed. They then participated in a fMRI study where inside the scanner they viewed videos of the eight individuals performing a simple goal-directed action (grasping and bringing a water bottle to the lips) or a video of them sitting behind a table (control condition). Each of these study components is described in detail below.

### Pre-Scan Training

All subjects participated in a training session prior to the scanning session. We modeled our approach after a previous study on social emotions that also consisted of a long and in-depth training session followed by a scanning session [30]. This approach allows participants to build a rich, emotional understanding of the images and stories.

During this session, participants were familiarized with eight individuals (“targets”) through the use of photos and corresponding fictional biographies. Half of these targets held strongly anti-Semitic beliefs. The targets’ biographies were created by searching for biographies and testimonials found in various media, and by combining facts and quotes into profiles that were one thousand words in length. Stories were constructed to have parallel structure and elements–for instance, each target story detailed events in childhood that shaped their belief system, and each story described role models that shaped the target’s behavior. Four of the stories involved targets from likable backgrounds, and the other four stories involved targets from dislikable backgrounds. Specifically, the eight biographies were divided up as follows: two never had an association with a neo-Nazi group; two began their lives normally, but then later joined a Neo-Nazi group; two began their lives in Neo-Nazi groups but later chose a life outside these groups; and lastly, two began their lives in Neo-Nazi groups and remained in these groups. Likable and dislikable stories were defined by group membership of the target at the end of the biography (e.g. a target that began their life as a Neo-Nazi, but left the group by the end of the biography was considered likable).

Participants were instructed to reflect on how they felt about the targets at the end of the biography. After each biography, participants filled out a brief questionnaire regarding how much they liked the target, how much time they would like to spend with the target, and how much they thought the target would like them. Participants were also asked to identify each of the targets and recount details of the target’s story as a way of ensuring that all the targets were equally and accurately remembered. Additionally, prior to scanning, they were shown the action and control video clips performed by the same actors that they would view in the scanner, for familiarization.

To account for possible sex differences, in each condition (likable targets and dislikable targets) there were two male targets and two female targets for each type of story. Likable targets were characterized as being open-minded, intelligent and positive in nature. By contrast, dislikable targets were strongly racist and anti-Semitic, uninterested in education, cynical of the world and expressly ungrateful for gifts bestowed to them. To accurately assess the likability of each target, in a separate behavioral pilot study, we asked 26 college students from the university subject pool to rate the target stories. Participants in this pilot study labeled the anti-Semitic, dislikable targets as significantly less likable than their counterparts (*t*(25) = −22.744, p<.000001). Additionally, to control for physical appearance of the actors, the pairing of stories and actors was counterbalanced. No significant differences were found between the likable targets (*t*(12) = 2.02, p>.05), or the dislikable targets (*t*(12) = 1.064, p>.05) of different versions.

### Stimuli Used in fMRI

**Figure 1:**
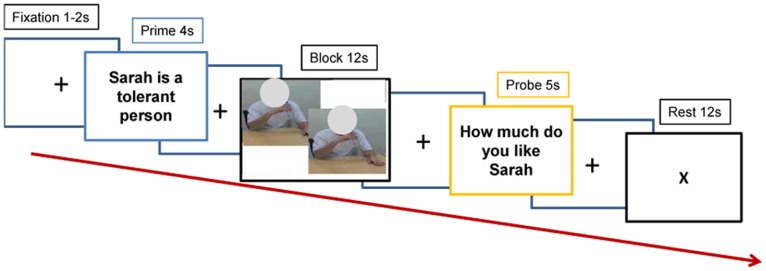
Schematic illustration of experimental design. Each trial began with a one sentence reminder of each target to be viewed in that given trial. A 1-s still photo from the first frame of the video clip preceded the action clip, which was shown twice, back-to-back. The action clips were followed by a question about how much the subject liked the viewed targets on a Likert scale from 1 to 4. The trial ended with a 12-second rest period that consisted of a centered “X” on a blank background screen. Circles used in the manuscript to protect the actor’s identity.

Inside the scanner, participants viewed 2-s movie clips (shown twice, back-to-back) of the target reaching for, grasping, and bringing a water bottle to their lips with the right hand (see Figure 1 for experimental design). Half of the clips displayed likable targets performing the action, and half displayed dislikable people performing the identical action. A 1-s still photo from the first frame of the video clip preceded the action clip in order for the participant to adequately process the target’s identity. As a control condition, clips of the same length, with the 1-s preceding still photo, were shown in which the target sat still with their right hand resting near the water bottle. Targets maintained neutral affect while performing actions. All actors were between 18–30 years of age to match the ages of the targets.

### Task Design and Procedure

- **fMRI of Action Observation.** The video clips were presented with MATLAB [31](The Mathworks, Inc., Natrick, MA), using the freely available Psychophysics Toolbox Version 3 software [32]. The visual stimuli were projected onto a rear projection screen at the end of the scanner bore which subjects viewed through a mirror mounted on the head coil. Participants were instructed to reflect on their feelings about the target while they viewed the movie clips during scanning.

A block design was used where two likable or two unlikable targets were presented together in each block. Trial blocks were preceded by a cue screen that presented two sentences serving as reminders for the participant of which targets they were about to view (e.g., In the case of likable targets: “Stephanie is a musician in New York. Julie wanted to raise her son to have an open mind.”). The cue screen was followed by a fixation cross (jittered 1–2-s), after which the clips were played for 12-s. After the clips were presented, a probe screen (5-s) followed asking the subjects to rate how much they liked the people they just viewed on a scale of 1 to 4, with 4 being “like very much.” The probe screen was followed by a 12-s rest condition. The presentation order of the block conditions was a pseudo-random, counterbalanced order to control for 1-back presentation history [30]. Each functional run consisted of ten blocks total, and there were three total functional runs conducted.

- **Behavioral Measures.** Prior to scanning, participants completed an ethnicity scale [29], a group membership scale based upon Schmitt et al. (2002) [33], the Interpersonal Reactivity Index (IRI) [34], the Brief Mood Introspection Scale (BMIS) [35], as well as likability ratings of all the targets after the completion of each biography. Further information on these scales and correlations between scores on these questionnaires and classification accuracy are reported in the Supporting Materials (Figure S1 and Figure S2). At the conclusion of the scanning session, participants were interviewed and debriefed. Interviews followed a script to identify how the participants felt about the targets when they viewed them in the scanner in addition to how well they remembered the targets. Participants’ responses were recorded during the interview. After the interview, we informed them to the purpose of the study, as well as revealing that the targets were actors.

#### Image Acquisition

Images were acquired with a 3-Tesla Siemens MAGNETON Trio System in the Dornsife Cognitive Neuroscience Imaging Center at the University of Southern California. Three functional runs, one anatomical MPRAGE, and one T2 weighted image was acquired for each subject.

Structural T_1_-weighted magnetization-prepared rapid gradient echo (MPRAGE) images were acquired (TR = 1,950 ms, TE = 30 ms, 224×256×176 matrix, 154 slices). 154 volumes of echo-planar volumes were acquired continuously with 37 slices per volume, and with the following parameters: TR = 2000 ms, TE = 30 ms, flip angle = 90°, 64×64 matrix with a spatial resolution of 3.5×3.5×3.5 mm, and interslice time = 54 ms, with no slice gap.

### Data Analysis

- **Multivariate Pattern Analysis.** Multivariate pattern analysis is a technique that uses machine learning algorithms to discriminate between neural activity patterns that differ between experimental conditions and/or stimuli types [24], [26], [36], [37]. While univariate fMRI data analysis techniques analyze each voxel’s activity individually, multivariate pattern analysis examines activity across several voxels together, allowing an examination of the spatial distribution of activity in a given region [24], [36].

MVPA was performed using the PyMVPA software package [38], implementing a linear support vector machine from LibSVM (http://www.csie.ntu.edu.tw/~cjlin/libsvm/). For each subject, data from the 3 functional runs were concatenated and motion corrected to the middle volume of the entire time series using FSL (FMRIB’s Software Library, http://www.fmrib.ox.ac.uk/fsl/index.html) and were then linearly detrended and converted to Z scores by run.

Given that the design of the experiment was a block design, we focused the MVPA analysis on the data from the entire duration of the 12-s blocks. With a 2-s TR, that provided six brain volumes per condition block for analysis, which were analyzed separately. To account for the delay in the hemodynamic response, we included all volumes from six seconds after the onset of each block to six seconds after the end of each block. Six regions of interest were defined based on regions previously implicated in action observation: bilateral pars opercularis, bilateral pars triangularis, and bilateral inferior parietal lobule (IPL). Pars opercularis and pars triangularis regions of interest were defined using the Harvard-Oxford cortical atlas, which is included with FSL, with a probability threshold of 70%. The left and right IPL were defined separately, using the Juelich cortical atlas, also included with FSL, with probability threshold of 85%. Each region of interest was then warped from standard space into each individual subject’s functional space. In each ROI, we performed 2 different types of classification. We performed a 2-way discrimination between the following conditions: likable individuals performing an action (Action Like) and dislikable individuals performing an action (Action Dislike). In addition, a 2-way discrimination between likable individuals in the control condition (Control Like) and dislikable individuals in the control condition (Control Dislike) was performed. We also performed 2 additional types of exploratory classifications, the results of which are reported in the Supporting Materials. A leave-one-out cross-validation approach was implemented where the classifier was trained on two functional runs and tested on the remaining run for each step of the cross-validation. The outcome of each step is classifier accuracy (performance), which was determined by dividing the number of correct classifier guesses by the number of test trials. Since we had 3 functional runs, cross-validation was repeated three times, and the accuracy results of the classifier for each step were then averaged together. In order to test the statistical significance of the results, a one sample t-test was conducted for each ROI across subjects, using the chance level as the test value, to test whether the sample’s classifier accuracy was significantly above chance.

In addition to the ROI analysis, we also performed a whole brain spherical searchlight analysis to investigate whether any regions falling just outside our regions of interest would have above chance classifier accuracy [27], [36]. A searchlight radius of 4 voxels (50 voxel clusters) was selected. In this method, multiple multivariate pattern classifications were carried out for a sphere centered on each voxel in the brain. The same linear SVM classifier with a leave-one-out cross validation was used for this analysis producing accuracy maps for each subject. Each subject’s map was warped into the Montreal Neurological Institute (MNI) standard space. In order to test for statistical significance of the resulting searchlight accuracy maps, t-tests were used to compare the 17 subjects’ accuracy maps to chance value (e.g. 0.5 for the Action Like and Action Dislike analysis). The results were then FDR corrected for multiple comparisons, p<0.05.

- **Univariate Analysis.** All fMRI and structural MRI pre-processing were completed using BrainVoyager [39]. Anatomical images were normalized to standard space with the following steps: inhomogeneity correction, alignment to ACPC space, and then conformation to Talairach space [40]. The fMRI data were first preprocessed for slice scan time correction using cubic spline interpolation in ascending, interleaved order, after which 3D motion correction was performed along six axes. The second run of the session was coregistered manually to the MPRAGE anatomical volume and transformed into Talairach space. After motion correction, the runs were aligned to the second functional run from the session. The data were then smoothed with an 8 mm FWHM 3d Gaussian kernel and temporally filtered using a high-pass filter.

**Figure 2:**
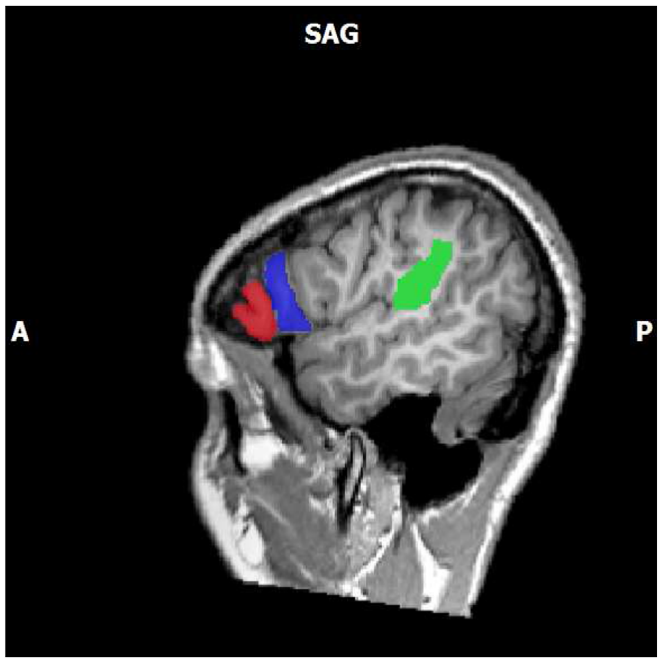
A priori defined regions of interest. ROIs were drawn on Talairach transformed MPRAGE images by hand using BrainVoyager. Limits were derived using Damasio (2005) for all regions. Pars triangularis (IFG; p.t.; shown in red), pars opercularis (IFG, p.o.; shown in blue), and inferior parietal lobule (IPL; shown in green).

At the first level of analysis, a general linear model was applied using the canonical hemodynamic response function (HRF). Six explanatory variables were included in the model: prime, likable targets performing an action, disliked targets performing an action, likable target action control, dislikable target action control, and probe. Minor head movements along six axes that took place during the runs were included as regressors of no interest into the design matrix to reduce motion artifacts. At the second level of analysis, the individual runs were included in a random effects (RFX) general linear model (GLM) analysis using both a region of interest (ROI) and a whole brain analysis respectively. To more directly measure activity within areas associated with action observation, regions of interest were hand-drawn and the activity inside was averaged across the runs to provide measures of contrast between conditions. ROIs were hand-drawn for each subject based upon anatomical boundaries detailed in [41] (See Figure 2 for locations; see Table S1 for boundaries). The regions were drawn on each subject’s Talairach-transformed anatomical images using BrainVoyager’s hand drawing tool. Analysis took the form of a random effects analysis of the comparison of the averaged time course of the functional BOLD data contained in the individual ROIs using a p-value of less than.05. Analyses were done using a t-test on the baseline corrected beta value difference between comparisons of pairs of conditions (e.g., Action Dislike vs Action Like). Lastly, results from the searchlight analysis that were located outside the a priori defined regions of interest were used as a mask in a univariate analysis.

## Results

### (a) Behavioral

Participants rated the dislikable (neo-Nazi) targets as significantly less likable than the likable targets (controls) (*t* (16) = −19.755, p<0.0001). In the pre-scan training session, participants rated the neo-Nazi targets less likable, as well as less apt to like, and less likely to spend time with the subject himself. Immediately following each block during the scanning session, participants were also asked to rate how much they liked the people performing the actions on a scale of 1–4, with 1 being you don’t like them at all, and 4 being you like them a lot. These ratings indicated that dislikable (neo-Nazi) targets were rated as significantly less likable by participants as compared to the likable targets (p<0.000001).

### (b) Multivariate Pattern Analysis (MVPA)

The two-way discrimination between Action Like and Action Dislike did not produce statistically significant results in any of the ROIs, nor did the two-way discrimination between Control Like and Control Dislike (see Table 1).

**Table 1:** Region of Interest Classification Accuracies.

*Discrimination* *Conditions*	*ROI*	*Chance Level*	*% Accuracy*	*p-value*
Action Like,Action Dislike	IFG, p.o., left	50%	50%	.870
	IFG, p.o, right		50%	.916
	IFG, p.t., left		49%	.861
	IFG, p.t., right		54%	.150
	IPL, left		49%	.578
	IPL, right		48%	.234
Control Like,Control Dislike	IFG, p.o., left	50%	52%	.447
	IFG, p.o, right		51%	.506
	IFG, p.t., left		53%	.213
	IFG, p.t., right		54%	.209
	IPL, left		50%	.819
	IPL, right		50%	.981

*Note.* IFG, p.o. = Inferior Frontal Gyrus, pars opercularis; IFG, p.t. = inferior frontal gyrus, pars triangularis; IPL = inferior parietal lobule. * indicates statistical significance, p<0.05.

**Figure 3:**
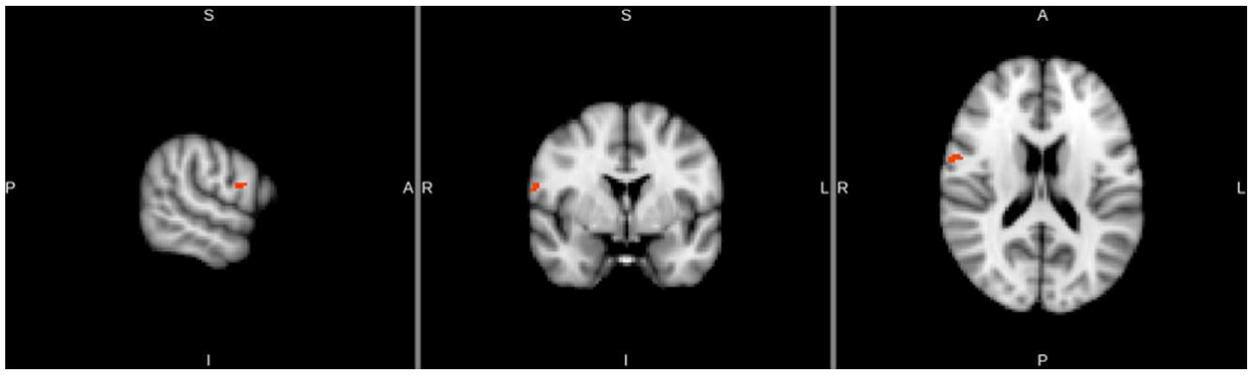
Searchlight analysis results. Classification accuracy in the right vPMC across subjects that survived FDR correction for multiple comparisons (FDR, p<.05) is displayed. Peak voxel coordinate in MNI space is (15, 64, 46).

**Figure 4:**
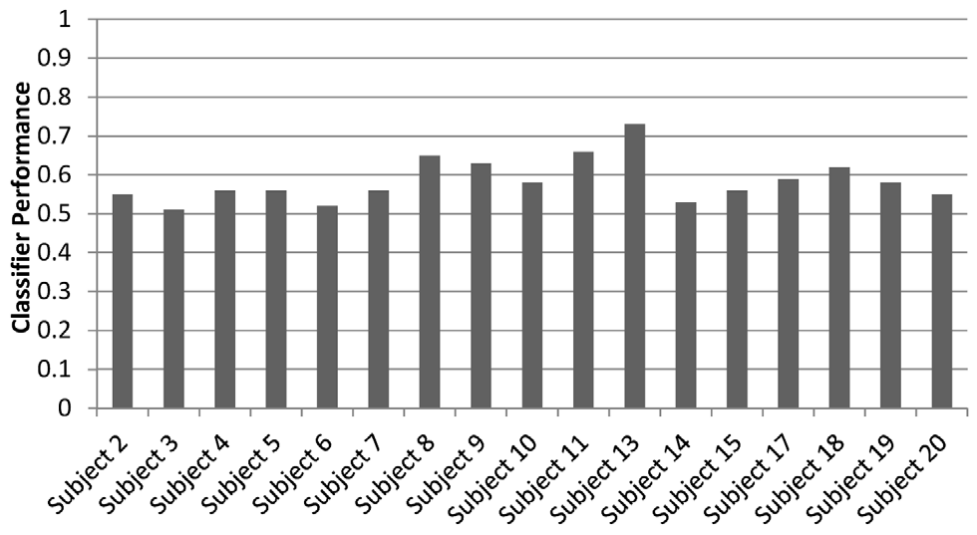
Searchlight classifier performance within right vPMC for individual subjects. Each bar represents searchlight peak classifier performance within the right vPMC in an individual subject for the Action-Like and Action-Dislike discrimination analysis. Chance performance is 0.5.

The same discriminations were performed in a whole brain searchlight analysis. The two-way discrimination between Action Like and Action Dislike revealed a significant classifier accuracy cluster in the right vPMC (*p*<.05, FDR corrected for multiple comparisons) just outside of the right pars opercularis ROI (See Figure 4). Classification accuracy in this region was above chance level for all participants, with the average of the peak classification accuracy across subjects being 58.47% (Figure 3 for significant cluster location, see Figure 4 for classification accuracy per subject). The two-way discrimination between Control Like and Control Dislike did not reveal any significant clusters in motor-related regions.

### (c) Univariate Analysis

**Figure 5:**
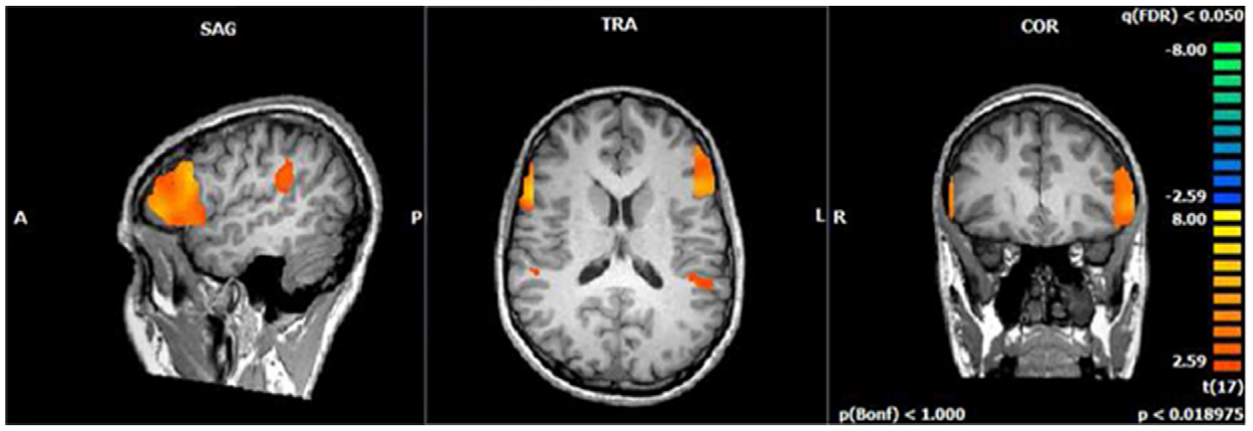
Region of Interest univariate analysis results. Differences in activation while watching all action clips compared with rest condition (Action Observation > Rest) are displayed. The *a priori* defined ROIs (IFG, pars triangularis; IFG, pars opercularis; IPL) were used as pre-threshold masks in the analysis. Results are displayed at p<0.01 (FDR corrected).

For the comparison between all action conditions (Likable and Dislikable) and rest, all action observation ROIs were significantly more active for action conditions than for the rest conditions: left and right IFG, pars opercularis, left and right IFG, pars triangularis, and left and right IPL (see Figure 5; see Supporting Materials for whole-brain analysis). There were no significant differences for any other comparisons within the ROIs. Lastly, when the vPMC significant cluster from the searchlight analysis was used as an ROI, no significant differences were found for the comparison between Action Like and Action Dislike.

## Discussion

We set out to investigate whether motor-related regions involved in action observation are modulated by a social factor, interpersonal liking derived from social group membership. Based on previous research that indicates these regions are affected by socially relevant factors, we predicted that they would have differential neural signatures during the viewing of a likable person performing an action as opposed to a dislikable person performing the same action. The whole-brain searchlight classifier found above-chance classification in the right vPMC discriminating between watching a person you like perform an action and watching a person you dislike perform an action, in line with our predictions. Additionally, these results were specific to action observation, as the classification of the control conditions of like and dislike demonstrated no significant difference. By contrast, the a priori regions of interest did not display differences in level of neural activity, as measured with univariate methods. Further, when this significant searchlight classifier cluster was used to mask the comparison of Action Like and Action Dislike in a univariate analysis, no significant differences were found. We believe this suggests that the classifier was able to extract a difference in activation patterns where the univariate analysis failed to detect an effect.

Our results are novel in that they demonstrate for the first time these motor-related regions are modulated during action observation by interpersonal liking derived from social group membership, a higher level classification which is not based on visual cues of group membership. Neuroimaging studies that have shown differential neural activity for group membership have typically used race as the defining factor for groups [23], [42]. Additionally, previous studies of action observation have indicated differential corticospinal excitability and differential BOLD activity levels in motor-related neural regions for perceived differences in social group membership, although these studies have also relied on visual cues of physical differences to indicate social group membership status (i.e. race). These past results are not surprising, given the body of literature that suggests differential activity in these action observation-related neural regions for self-other distinction [14], [43], as well as for individuals who are physically dissimilar from the self [9]. In our study, the likable targets, dislikable targets, and participants themselves were physically similar to each other to rule out effects of physical cues to group membership. Here, we begin to tease apart this complex relationship by taking advantage of two social groups that appear physically similar, but consider each other as dislikable outgroups. Similar to our findings, a more abstract definition of group membership (i.e. political party affiliation) was found to have an effect on the perception of touch [44]. Together, these results indicate that it may indeed be the higher level abstraction of group membership, and not only differences in physical appearance, that affect basic sensory-motor processing.

Additionally, unlike previous studies, we address the more basic question of whether these socially relevant factors influence the perception of simple object directed actions, rather than higher level action understanding through communicative cultural gestures. Previous studies comparing the observation of actors of different races performing culture-specific gestures have shown differential corticospinal excitability and neural activity [5], [6]. While these studies are important in proving that social and cultural factors can influence motor representations of communicative and meaningful hand gestures, it is of interest to investigate whether these social factors solely influence neural processing for communicative gestures with cultural meaning, or whether their influence extends to non-cultural, object directed actions, as well. Our finding that the ventral premotor region in the right hemisphere displayed differential neural activity for the observation of simple object directed motor actions appears to support this latter notion.

In this study, the neural pattern of activity in the vPMC was modulated by interpersonal liking derived from social group membership. The right hemisphere has been suggested to be the hemisphere that plays a larger role in social and emotional processing in humans [45]–[47]. Although our visual stimuli were simple object-directed actions, the biographies of the targets were emotionally charged indicators of which social group the target belonged. The subjects were instructed to remember how they felt about the targets while viewing the video clips in the scanner, and they did, in fact, report feeling differently about how much they liked the different groups of targets. This demonstrates that the amount of interpersonal liking was modulated by group membership, and that this higher level processing may color the observation of actions, particularly in the right vPMC.

Overall, these results contribute to the longstanding evidence supporting the notion that perception of ingroup and outgroup members implicitly biases information processing in fundamental neural networks. Behaviorally, actions from outgroup members can be perceived and described as more negatively [48], [49], or judged as more slow [50], as opposed to ingroup members’ actions, implying that differential perception and processing of ingroup members and outgroup members may occur. Supporting behavioral findings, physiological and neural differences have also been found when perceiving ingroup and outgroup members [11], [23]. This study expands our knowledge of this phenomenon by indicating that these higher level cognitive constructs can affect perception, and specifically, can modulate sensory-motor processing.

It remains to be seen whether these differences in neural patterns of activity will persist during an action observation task when the disliked outgroup is physically similar to oneself, but it is not socially acceptable to express disdain for members of the social group. The main focus of this study was specifically to examine interpersonal liking as it is derived from social group membership, in a circumstance that is free from social stigma against disliking the outgroup member. An additional concern is social desirability, or the desire of the participant to give a response they believe the researchers want to hear. Although it is difficult to rule this out in the current study, it is worth noting that the subjects had quite visible negative reactions to the emotionally evocative stories, possibly suggesting that their feelings towards the targets corresponded to their negative ratings. Investigating the difference between likability for an individual who is disliked for a personal reason versus for a social reason is an interesting topic for a future study.

Our results indicate that socially relevant factors, such as interpersonal liking and group membership, can affect motor-related neural regions, those underlying action observation. Specifically, the right vPMC exhibits differential neural activity patterns during the observation of a likable person, as opposed to a dislikable person specifically during action observation. Our research confirms previous findings that activity in action observation related neural regions can be modulated by social group membership, and we extend this finding by removing any possible effects of physical differences between group members and expanding beyond communicative cultural gestures. Our data suggest that neural regions involved in action observation of simple goal-oriented actions are tuned to interpersonal liking derived from social group membership.

## Supporting Information

Figure S1 **Individual subject searchlight accuracy maps for Action Like-Action Dislike classification.** Crosshair is located in the vPMC cluster that was significant at the group level. All individual subject maps are warped into MNI space, and thresholded so that only regions showing above chance classification (greater than 50%) are shown.
(TIF)

Figure S2 **Whole-brain univariate analysis for all action versus rest.** Differences in whole-brain activation while watching all action clips compared with rest condition (Action Observation > Rest) are displayed. Results are displayed at p<0.05 (FDR corrected).
(TIF)

Figure S3 **Relationship between ethnicity scores and searchlight peak accuracy values from vPMC.** Correlation conducted across subjects, r = .46, p>.05, n = 15.
(TIF)

Figure S4 **Relationship between ethnicity scores and peak accuracy values for the right IPL.** Correlation conducted across subjects during the 4-class discrimination, r = .55, p<.05, n = 15.
(TIF)

Table S1 *Region of Interest Classification Accuracies.*
(DOCX)

Table S2 *Hand Drawn ROI Limits.*
(DOCX)
