# Supplementary figures and images for: Interpersonal Liking Modulates Motor-Related Neural Regions
Source: PLoS One. 2012 Oct 5;7(10):e46809. doi: 10.1371/journal.pone.0046809 (PMC3465281; doi:10.1371/journal.pone.0046809)

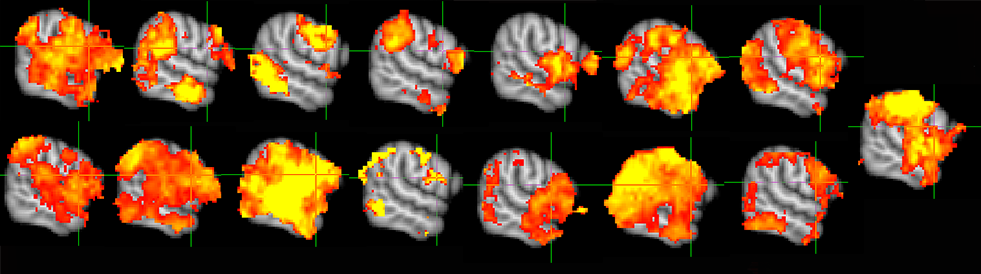

Supplement: Figure S1 — Individual subject searchlight accuracy maps for Action Like-Action Dislike classification. Crosshair is located in the vPMC cluster that was significant at the group level. All individual subject maps are warped into MNI space, and thresholded so that only regions showing above chance classification (greater than 50%) are shown. (TIF) [file pone.0046809.s001.tif]

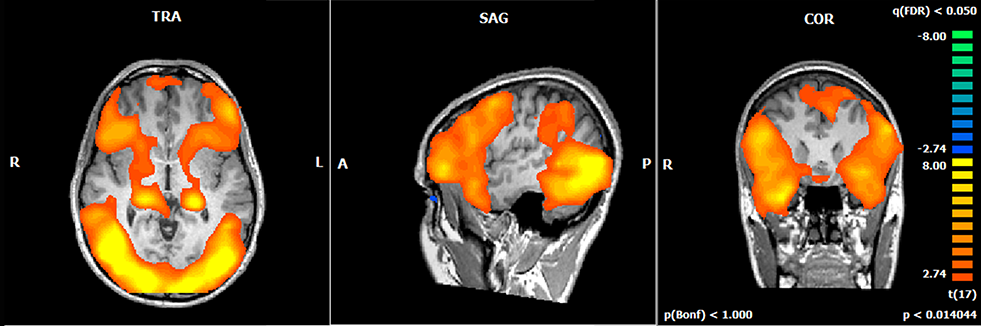

Supplement: Figure S2 — Whole-brain univariate analysis for all action versus rest. Differences in whole-brain activation while watching all action clips compared with rest condition (Action Observation > Rest) are displayed. Results are displayed at p<0.05 (FDR corrected). (TIF) [file pone.0046809.s002.tif]

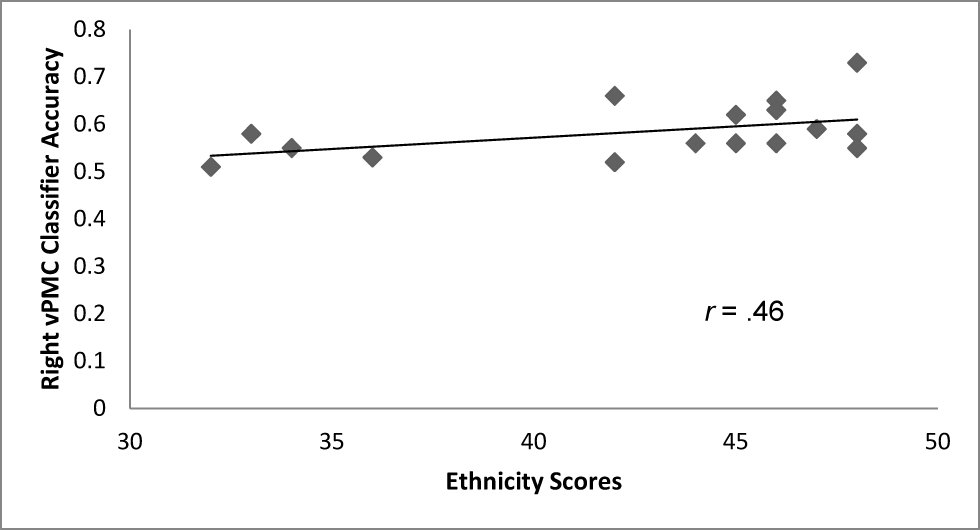

Supplement: Figure S3 — Relationship between ethnicity scores and searchlight peak accuracy values from vPMC. Correlation conducted across subjects, r = .46, p>.05, n = 15. (TIF) [file pone.0046809.s003.tif]

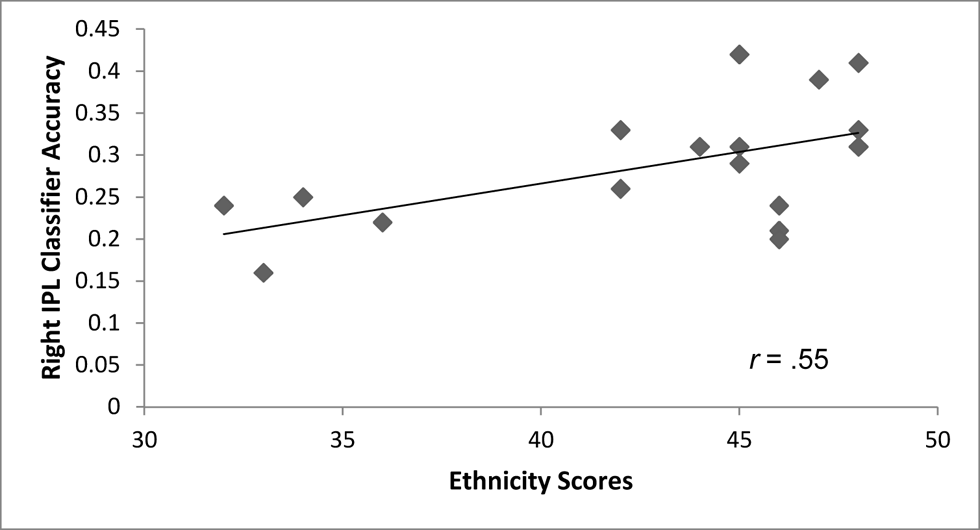

Supplement: Figure S4 — Relationship between ethnicity scores and peak accuracy values for the right IPL. Correlation conducted across subjects during the 4-class discrimination, r = .55, p<.05, n = 15. (TIF) [file pone.0046809.s004.tif]
