# Supplementary material for: Interpersonal Liking Modulates Motor-Related Neural Regions
Source: PLoS One. 2012 Oct 5;7(10):e46809. doi: 10.1371/journal.pone.0046809 (PMC3465281; doi:10.1371/journal.pone.0046809)
Supplement: Table S1 — Region of Interest Classification Accuracies. (DOCX) [file pone.0046809.s005.docx]

**Supporting Table 1.** *Region of Interest Classification Accuracies*

| *Discrimination Conditions* | *ROI* | *Chance Level* | *% Accuracy* | *p-value* |
| --- | --- | --- | --- | --- |
| Action Like, Action Dislike, Control Like, Control Dislike | IFG, p.o., left | 25% | 25% | .835 |
|  | IFG, p.o, right |  | 26% | .472 |
|  | IFG, p.t., left |  | 26% | .533 |
|  | IFG, p.t., right |  | 27% | .160 |
|  | IPL, left |  | 27% | .309 |
|  | IPL, right |  | 29% | .057 |
| Like (Action and Control), Dislike (Action and Control) | IFG, p.o., left | 50% | 52% | .161 |
|  | IFG, p.o, right |  | 54% | .065 |
|  | IFG, p.t., left |  | 55% | .008* |
|  | IFG, p.t., right |  | 51% | .439 |
|  | IPL, left |  | 52% | .202 |
|  | IPL, right |  | 52% | .047* |

*Note.* IFG, p.o. = Inferior Frontal Gyrus, pars opercularis; IFG, p.t. = inferior frontal gyrus, pars triangularis; IPL = inferior parietal lobule. * indicates statistical significance, p < 0.05.
