# Supplementary material for: Interpersonal Liking Modulates Motor-Related Neural Regions
Source: PLoS One. 2012 Oct 5;7(10):e46809. doi: 10.1371/journal.pone.0046809 (PMC3465281; doi:10.1371/journal.pone.0046809)
Supplement: Table S2 — Hand Drawn ROI Limits. (DOCX) [file pone.0046809.s006.docx]

***Supporting Table 2.*** *Hand Drawn ROI Limits*

| ROI | Anterior Limit | Posterior Limit | Inferior Limit | Superior Limit | Medial/Lateral Limit |
| --- | --- | --- | --- | --- | --- |
| **IFGpo** | Ascending branch of Sylvian fissure | Pre-central sulcus | Sylvian fissure | Inferior Frontal sulcus | Depth of the Ascending branch of Sylvian fissure |
| **IFGpt** | Inferior Frontal sulcus | Ascending branch of Sylvian fissure | Sylvian fissure | Inferior Frontal sulcus | Depth of the Ascending branch of Sylvian fissure |
| **IPL** | Inferior postcentral sulcus | Intersection of the parieto-occipital and intraparietal sulci | Lower branch of superior temporal sulcus | Depth of the intraparietal sulcus toward the insula | Depth of the intraparietal sulcus |

*Note.* IFGpo = Inferior Frontal Gyrus, pars opercularis; IFGpt = Inferior Frontal Gyrus, pars triangularis; IPL = Inferior Parietal Lobule.
